# Health-related quality of life in a general population sample in Kazakhstan and its sociodemographic and occupational determinants

**DOI:** 10.1186/s12955-021-01843-4

**Published:** 2021-08-21

**Authors:** Denis Vinnikov, Aizhan Raushanova, Zhanna Romanova, Zhangir Tulekov

**Affiliations:** 1 Faculty of Medicine and Healthcare, Al-Farabi Kazakh National University, Al-Farabi Avenue 71, 050040 Almaty, Kazakhstan; 2 Peoples’ Friendship University of Russia (RUDN University), Moscow, Russian Federation

**Keywords:** Quality of life, Occupational, Regression, Smoking

## Abstract

**Background:**

Health-related quality of life (HRQL) in the general population of Kazakhstan has never been characterized. We constructed this population-based study of the largest city in Kazakhstan, Almaty with the aim to quantitatively assess HRQL and ascertain whether occupation and lifestyle are associated with HRQL in this population.

**Methods:**

In a random sample (N = 1500) of general population in Almaty (median age 49 (interquartile range 28) years, 50% women), we collected data on demographics, socioeconomic status, lifestyle, lifetime occupational history and general HRQL using SF-8 instrument. The association of demographic and occupational predictors with HRQL was tested in multiple regression models.

**Results:**

No occupational associations were found for physical component score in the models adjusted for age, sex, income, cigarette and waterpipe smoking, electronic cigarette use, physical activity, alcohol and exposure to secondhand smoke. Ever being a manager (β − 1.63 (95% confidence interval (CI) − 2.92; − 0.34)), a welder (β − 5.11 (95% CI − 8.77; − 1.46)) and a secretary (β − 5.06 (95% CI − 8.56; − 1.56)) for one year or more was associated with poorer mental component score in the models adjusted for age, sex, income, cigarette smoking, physical activity and each other. Age, income and physical activity were independent predictors of both physical and mental components.

**Conclusions:**

Occupational history is associated with HRQL in the general population in Almaty, Kazakhstan, but the mechanism explaining this association should be further elucidated.

## Background

Health-related quality of life (HRQL) is a subjective perception of individual’s well-being resulting from health status. HRQL is believed to reflect an individual’s adaptation to a deficit or suffering from chronic conditions and it is largely explained by the presence and severity of diseases. Most often interpreted by two components, physical and mental component, HRQL serves an indicator in addition to standard ways of assessing medical interventions’ effect, since it is affected not only by health status, but social and even economic determinants.

HRQL has been assessed in various occupations and has been found to have some association with selected occupational groups, such as nurses [1] or welders [2]. As expected, HRQL in the former was associated with compassion fatigue and burnout [3], and that may be true for other occupations. In other occupations, HRQL was found to be linked to physical activity, as in police [4]; however, studies are not consistent in ascertaining vulnerable occupations, and occupations shown to be at risk in one region can exhibit protective effect in the other. Thus, drivers, agricultural and fishery workers demonstrating the worst HRQL in a large sample [5] do not differ from the general population in other studies. Because HRQL by definition reflects satisfaction and adaptation to one’s health with given set of chronic or acute diseases, whether it is occupation that deteriorates health and HRQL or, alternatively, people with baseline worse health and HRQL choose selected occupations, remains unclear.

General HRQL in Kazakhstan general population has never been characterized, nor has the effect of healthy behavior or lifestyle been included in such analyses. Furthermore, almost nothing is known about the association of lifetime occupations and HRQL in this population. We hypothesized that smoking, alcohol, regular physical activity and occupational history in Kazakhstani population may impact HRQL. Therefore, we constructed this population-based study of the largest city in Kazakhstan, Almaty with the aim to quantitatively assess eight widely used domains of HRQL and ascertain whether occupation and lifestyle are associated with HRQL in this population.

## Methods

The study was approved by the Committee on Bioethics of Al-Farabi Kazakh National University. This was a population-based study in the largest Kazakhstan cities, Almaty (population around 2 million), and the overall methodology was described earlier [6]. The primary analysis of this study included testing the association of lifetime occupational history with chronic obstructive pulmonary disease and was published elsewhere [6]. However, HRQL described in this presentation, was the secondary variable of interest and was collected in all participants. Data were collected during 4 months of 2019, March through June.

In Kazakhstan, each policlinic (family medical center) offers primary level services to all population within its coverage, including adults and children. We have used data of one of Almaty central policlinics with the total population coverage 85,000+, including 66,000+ adults, and randomized the population to eventually form a sample of 1500 adults (18 years and older). Because study participants were randomly selected from the total population register, they did not represent the strata of people with medical conditions or symptoms; instead, they represented the general population of the territory covered by the polyclinic. Included subjects were invited by the nurse to attend the medical facility for interview any time during the day. Following introduction and informed consent acquisition, research team interviewed each subject face-to-face offering a structure questionnaire. The questionnaire included demographic patient’s data, such as name, date of birth, sex, telephone number, overall work duration, highest attained education (secondary school, high school, college, university or academic degree), and a total household income (100,000 tenge and less, 101,000 to 500,000 tenge or 501,000 tenge or more a month). We also asked to provide exact permanent residence address.

Smoking status was ascertained via four questions, categorizing subjects to never-, ex-, current occasional and current daily cigarette smokers. Daily smokers were asked to provide information on the number of smoked cigarettes per day and years smoked. In this section, we also asked about waterpipe and electronic cigarette smoking, exposure to secondhand smoke in the workplace or at home (yes/no), regular exercise at least 3 times a week (yes/no), and finally alcohol use intensity. The latter question offered 4 options to answer from ‘never’ to ‘using once a week or more often’.

In the current presentation, we evaluated generic HRQL using the short version of SF questionnaire, SF-8, validated Russian version, in which HRQL was assessed for the last 4 weeks. The tool consisted of eight questions, corresponding to eight major HRQL domains, including general health (GH), physical and social functioning (PF and SF), bodily pain (BP), role physical (RP), role emotional (RE), mental health (MH) and vitality (VT) and was self-administered. Each domain was scored using the manual for SF-8 tool [7]. We analyzed each domain as a dependent variable separately, and also calculated physical and mental component scores (PCS and MCS). Those were treated as continuous variables in all analyses. In addition, we collected detailed occupational history from each subject. Any position held by a subject for a year or more was recorded with the corresponding years of service in each position. Similar occupations like manager, director or administrator were coded as one occupation. Furthermore, we used a set of 29 questions from a questionnaire used in previously published studies [8] to ask about specific occupational exposures, such as metal aerosol, carbon powder, ceramics, etc. That questionnaire also ascertained duration of exposure and hours worked per week. Exposures lasting one year and more were further analyzed as specific occupational exposures in addition to the occupations held.

We analyzed distribution in all included variables using Shapiro–Wilk test and found no normality; therefore, we used non-parametric tests for between-group comparisons in this analysis. The scores of each of eight domains and the summary scores (PCS and MCS) are described as their 5th, 25th, 50th (median), 75th and 95th percentiles. In the univariate analyses, we compared either two selected groups with each other, such as males versus females, or used contingency tables for multiple-group variables, such as age, stratified into categories. *P* values were calculated for all χ^2^ tests. Continuous variables in the univariate analyses were tested using Mann–Whitney test, whereas χ^2^ test was the alternative method for binary variables. We then used regression models to test sociodemographic and lifestyle predictors of PCS and MCS separately. At this stage, we applied simple regression models to select significant predictors with *P* < 0.05. In addition, we tested each occupation against PCS and MCS scores separately as continuous variables in simple regression models and reported betas with their corresponding 95% confidence intervals (CIs) and *P* values. These analyses only included occupations with the reported prevalence of 1% or more. For this, we created a number of binary variables, coded 0 (no history) or 1 (with occupational history in this occupation). Accordingly, participants with no occupational history were coded 0 in all such variables. Less prevalent occupations were excluded because of insufficient power for analyses. Occupations with significant associations with PCS or MCS in these simple regression models were then tested in multiple models with sociodemographic variables (separate set of variables for PCS and MCS). The skewness of PCS (− 0.505) and MCS (− 0.652) were considered mild and allowed to used multiple regression models for the analysis. In such analysis we also looked for multicollinearity using correlation matrix, but none of tested predictors was excluded because we found no variables with moderate or high correlation with each other. Significant predictors in adjusted for sociodemographic variables were then tested against each other in a fully adjusted model. All tests were run in NCSS 2020 (Utah, USA).

## Results

Half of the sample were women (N = 752, 50%). The median age was 49 (interquartile range 28) years; 1% had an academic degree, 51% had a university degree, 28% were college graduates, 19% had only high school and the remaining 1% only had secondary school education (Table 1). Only 2% had a household income exceeding 501,000 tenge (1290 United States dollars) a month. Forty-one percent of subjects never smoked cigarettes, 28% smoked in the past and 31% were current smokers. Sex affected almost all included sociodemographic and lifestyle determinants. Thus, more women had higher education, smaller income, demonstrated smaller prevalence of all studied tobacco products and more women were never alcohol users. The least median score of eight studied HRQL domains was 38.4 (GH), the highest—53.4 (BP) (Table 2).

**Table 1:** Demographic, socioeconomic and lifestyle determinants of the studied sample

Variable	Overall	Men	Women
N	1500 (100)	748 (50)	752 (50)
Age, years*	49 (28)	45 (29)	54 (23)
Education, N (%)*			
Secondary school	15 (1)	8 (1)	7 (1)
High school	282 (19)	179 (24)	103 (14)
College	416 (28)	173 (23)	243 (32)
University	764 (51)	373 (50)	391 (52)
Academic degree	23 (1)	15 (2)	8 (1)
Monthly income, N (%)*			
10,000 tenge or less	535 (36)	242 (32)	293 (39)
100,000–500,000 tenge	925 (62)	485 (65)	440 (59)
More than 500,000 tenge	36 (2)	20 (3)	16 (2)
Cigarette smoking, N (%)*			
Never	620 (41)	152 (20)	469 (62)
Former	418 (28)	269 (36)	149 (20)
Current	462 (31)	327 (44)	134 (18)
Current waterpipe smoking, N (%)*	143 (10)	109 (15)	34 (5)
Current electronic cigarette use, N (%)*	45 (3)	34 (5)	11 (1)
Exposure to secondhand smoke, N (%)*	610 (41)	331 (44)	279 (37)
Regular physical activity, N (%)	462 (31)	248 (33)	214 (28)
Alcohol use, N (%)*			
Never	746 (50)	310 (41)	436 (58)
Small amount sometimes	480 (32)	233 (31)	247 (33)
Moderate amount sometimes	185 (12)	132 (18)	53 (7)
At least once a week	89 (6)	73 (10)	16 (2)

*SHS* secondhand smoke; pairwise comparisons are from χ^2^ test or Mann–Whitney U-test. Tests of education, household income, cigarette smoking and alcohol use are from 2 * 5 2 * 3 and 2 * 4 tests; * – *p* < 0.05

**Table 2:** Eight main domains and composite scores (PCS and MCS) in the general population of Almaty

Domain or composite score	5th percentile	25th percentile	50th percentile	75th percentile	95th percentile
Physical functioning (RF)	30.3	40.1	48.3	48.3	54.1
Social functioning (SF)	29.5	49.5	52.3	52.3	52.3
Role physical (RP)	28.3	38.7	54	54	54
Role emotional (RE)	29.3	38.1	52.4	52.4	52.4
Mental health (MH)	31.6	41.5	49.6	56.8	56.8
Vitality (VT)	35.8	45.2	45.2	55.6	61.8
Bodily pain (BP)	40.1	47.7	53.4	60.8	60.8
General health (GH)	38.4	38.4	38.4	46.4	59.5
Physical component score (PCS)	40.7	50.9	57.7	63.1	68.0
Mental component score (MCS)	41.1	51.8	59.5	65.7	69.7

In the univariate analysis, both physical and mental components were associated with age and sex (Table 3). PCS had more advanced decline with age compared with mental component. Women had significantly worse HRQL in both domains. College of university education did not affect any HRQL component, whereas monthly income had a positive association with both physical and mental scores, but the effect was greater for PCS. Subjects smoking cigarettes, electronic cigarettes or using waterpipe had a significantly greater PCS score, apparently because they were yet healthy enough to smoke. Cigarette smoking, but not waterpipe use of electronic cigarette smoking, was also associated with mental component. Regular physical activity positively affected both HRQL domains. Alcohol use was associated with higher physical component of HRQL, but not with mental.

**Table 3:** PCS and MCS and their demographic, socioeconomic and lifestyle predictors in the univariate analyses

Variable	PCS			MCS		
	Beta	Beta 95% CI	*p*	Beta	Beta 95% CI	*p*
Age	− 0.20	− 0.23; − 0.17	< 0.001	− 0.07	− 0.09; − 0.04	< 0.001
Sex (reference—male)	− 2.19	− 3.05; − 1.34	< 0.001	− 3.38	− 4.30; − 2.46	< 0.001
Education (reference—secondary or high school)	− 0.49	− 1.57; 0.59	0.38	− 0.85	− 2.02; 0.36	0.15
Monthly income (reference—below 10,000 tenge)	3.20	2.31; 4.08	< 0.001	1.15	0.17; 2.12	< 0.05
Current cigarette smoking (reference—never or former)	1.50	0.52; 2.48	< 0.01	1.31	0.25; 2.37	< 0.05
Exposure to SHS (reference—no)	2.40	1.54; 3.27	< 0.001	0.82	− 0.13; 1.77	0.09
Waterpipe smoking (reference—no)	5.44	4.00; 6.88	< 0.001	1.48	− 0.11; 3.06	0.07
Electronic cigarette use (reference—no)	4.67	2.16; 7.19	< 0.001	1.67	− 1.05; 4.43	0.23
Regular physical activity (reference—no)	2.48	1.55; 3.40	< 0.001	2.27	1.26; 3.27	< 0.001
Alcohol use (reference—never)	1.48	0.62; 2.34	< 0.001	− 0.04	− 0.97; 0.90	0.94

*PCS* physical component score, *MCS* mental component score, *SHS* secondhand smoke, *CI* confidence interval

We excluded occupations with the fraction 1% or below in the overall employment profile because of low power. These excluded occupations were expeditor, plumber, fueler, musician, filmmaker, controller, coddler, carpenter, pilot, pressman, pharmacist, geologist, turner, architect, interpreter, journalist, baker, gardener, psychologist, librarian, flight attendant, metallurgist, miner and mapmaker. A number of occupations were associated with the physical component of HRQL, but none of them retained significant association when adjusted for socioeconomic determinants or lifestyle (Model 1 in Table 4), indicating that not the occupation, but the underlying socioeconomic determinants and lifestyle predicted HRQL. Furthermore, such occupations as driver (N = 104, 6.9%), civil servant (N = 101, 6.7%), construction (N = 96, 6.4%), nurse (N = 82, 5.5%), businessman (N = 78, 5.2%), work in catering (N = 76, 5.1%), security officer (N = 58, 3.9%), foreman (N = 56, 3.7%), medical doctor (N = 41, 2.7%), military officer (N = 36, 2.4%), tailor (N = 33, 2.2%), mechanic (N = 31, 2.1%), police officer (N = 29, 1.9%), electrician (N = 27, 1.8%), machinist (N = 26, 1.7%), metal worker (N = 26, 1.7%), agricultural worker (N = 24, 1.6%), technologist (N = 22, 1.5%), artist (N = 17, 1.1%), hairdresser (N = 16, 1.1%), fitter (N = 16, 1.1%), lawyer (N = 17, 1.1%) or fitness trainer (N = 15, 1.0%), were not associated with either PCS and or MCS even in the crude models. In such an adjusted model, only age, income and physical activity could determine PCS. Of note, age and income had a negative association, whereas physical activity shower positive association. Of all included occupations, only lab technicians had some, but yet non-significant (*p* = 0.055) negative association with PCS in the models adjusted for socioeconomic determinants and lifestyle.

**Table 4:** Occupational determinants of HRQL in crude and adjusted models

Position	N (%)	PCS		MCS	
		Crude beta (95% CI)	Model 1 beta (95% CI)	Crude beta (95% CI)	Model 2 beta (95% CI)
Teacher or faculty	386 (25.7)	− 1.84 (− 2.83; − 0.86)	NS	NS	–
Manager	222 (14.8)	1.84 (0.63; 3.05)	NS	− 1.46 (− 2.77; − 0.14)	− 1.66 (− 2.95; − 0.36)
Economist	143 (9.5)	− 2.32 (− 3.78; − 0.85)	NS	− 2.05 (− 3.63; − 0.46)	NS
Sales person	143 (9.5)	NS	–	− 1.88 (− 3.47; − 0.29)	NS
Laborer	118 (7.9)	− 2.01 (− 3.61; − 0.41)	NS	NS	–
Engineer	111 (7.4)	− 1.67 (− 3.31; − 0.02)	NS	NS	–
Kindergarten teacher	54 (3.6)	NS	–	− 3.37 (− 5.87; − 0.87)	NS
Secretary	26 (1.7)	NS	–	− 6.52 (− 10.09; − 2.96)	− 5.18 (− 8.70; − 1.67)
Welder	24 (1.6)	− 3.67 (− 7.10; − 0.24)	NS	− 3.92 (− 7.64; − 0.20)	− 5.03 (− 8.70; − 1.36)
Information technology specialist	22 (1.5)	NS	–	4.04 (0.16; 7.92)	NS
Lab technician	20 (1.3)	− 5.65 (− 9.40; − 1.91)	NS	NS	–

Occupations with non-significant associations in crude models are not included. *NS* non-significant, *PCS* physical component score, *MCS* mental component score. Model 1: adjusted for age, sex, monthly income, cigarette and electronic cigarette smoking, waterpipe use, physical activity, alcohol use and exposure to SHS. Model 2: adjusted for age, sex, monthly income, cigarette smoking and physical activity

In a univariate analysis, such occupations as economist, manager, kindergarten teacher, sales person, welder and secretary were associated with poorer mental component of HRQL, but working as an IT specialist showed an opposite association. When adjusted for age, sex, monthly income, cigarette smoking and physical activity, only managers, welders and secretaries showed significantly worse MCS, more pronounced for welders and secretaries (Model 2 in Table 4). In a fully adjusted model, when significantly associated with HRQL occupations (manager, welder and secretary) were adjusted not only for age, sex, income and physical activity, but also for each other, they all remained significantly associated with MCS, assuming that such association was independent of each included occupation. Thus, in such fully adjusted model, ever being a manager reduced the mental component of HRQL by 1.63 (95% CI − 2.92; − 0.34) points; a welder—by 5.11 (95% CI − 8.77; − 1.46) and a secretary—by 5.06 (95% CI − 8.56; − 1.56) points. With regard to specific exposures, in an adjusted for sociodemographic predictors model, exposure to irritant gases for more than a year negatively affected PCS (beta − 3.20 (95% CI − 5.99; − 0.42)), whereas exposure to grain or flour in the workplace was associated with poorer MCS (beta − 3.51 (95% CI − 6.72; − 0.29)).

## Discussion

This is the first summary of HRQL of Kazakhstan general population using SF-8 in a sample of 1500 residents of Almaty showing relatively low score in GH domain and high score in BP domain. We showed that age, low monthly income and female sex were the strongest negative predictors of both physical and mental HRQL components, even when lifetime occupations were considered. Furthermore, regular physical activity was associated with higher HRQL score in both domains in all adjusted models, whereas cigarette and electronic cigarette smoking along with waterpipe use and alcohol were associated with higher physical HRQL. Selected occupations, such as being a manager, a welder or a secretary negatively affected mental component of HRQL in Kazakhstan general population.

Our study elicited surprising outcomes of higher PCS and MCS score in smokers, which contrasts selected other population-based [5] studies, as well as studies in only working population [9]. We believe that smokers reported better HRQL because such subjects were healthy enough not to be concerned with adverse health effects and the resulting diseases of unhealthy behavior. Hence, those with severe chronic conditions, which result in worse HRQL, have either never initiated smoking or already quit because of their medical condition. Other lifestyle attributes, such as regular physical activity have been confirmed to improve HRQL in selected occupational groups [4] and general population [10, 11]. Of note, our results assume better both physical and mental component in those exercising regularly, consistent with other reports, in which physical activity was associated with better mental component as well [10].

Attempts to link occupational exposures to poorer HRQL have been undertaken in the studies of welders [2], former lead workers [12], firefighters [13], industrial workers [14, 15] and even coal-based sponge iron plant workers [16]. All these presentations confirmed negative associations of studied occupations with either physical or mental component of HRQL. Another group of studies tested whether selected exposures in the workplace were associated with lower general HRQL scores. For example, exposure to noise, industrial dust and stress had a negative effect on HRQL in the informal workers, whereas in formal workers the association was confirmed with regard to chemical substances, noise, sun, occupational stress, biological material and industrial dust [17]. In addition, studies also demonstrated that exposure to vapors, gases, dusts and fume in an industrialized area was associated with worse HRQL irrespective of chronic obstructive pulmonary disease [18]. The latter study concluded that negative association of exposure with HRQL may persist even after retirement.

Almost all studies listed above were limited to a shorter list of tested occupations or exposures. We aimed to embrace a wider list of occupations mentioned when filling the questionnaires by the general population in our sample. In total, there were 59 occupations listed, but the occupational association was only detected in managers, welders and secretaries adjusted for other significant sociodemographic predictors of HRQL, and only in MCS. Negative association of working as manager with HRQL was somewhat a surprising finding, because an earlier population-based study reported the opposite association. Albeit physiological explanation of these associations goes beyond the scope of the analysis, and, moreover, the underlying mechanisms would be hard to scrutinize, we hypothesize that selection of candidates for a manager position is subject to local cultural and traditional variations, and subjects with poorer health and resulting HRQL are more likely to work on managerial positions in Kazakhstan. We believe that same relates to secretaries. As for welders, our report is consistent with previously sporadic presentations showing worse HRQL in welders.

The limitations of this study should be noted. Firstly, cross-sectional design of this analysis did now allow to test and verify causality in the associations we had identified. Secondly, the study was limited to Almaty only, whereas other cities in Kazakhstan with smaller population may have different occupational history of population due to alternate correlation of people employed for industry, agriculture and government sector. Furthermore, transition from the Soviet planned economy to the current model has profoundly changed the profile of the population employment with dramatic shift from industrial position to sales. HRQL quantitative assessment during the times of more people working in the industry in the past for comparative purposes would have helped better understand the association of occupational exposures with HRQL. Thirdly, we only used general QL tool, which cannot detect more specific domains of QL, attributable to chronic diseases and conditions, also limiting HRQL. Finally, we did not consider the full range of morbidity profile of participants, including chronic diseases, such as coronary artery disease or asthma, which may have also impacted HRQL.

The findings of this population-based study have distinct implications for public health and service. The income remains one of the most powerful determinants of HRQL, irrespective of age, smoking, physical activity, occupation, sex and exposure to secondhand smoke. This mandates further governmental effort to reduce poverty, but not only to improve healthcare, enhance primary and earlier secondary prevention of diseases which are known to lessen HRQL. Moreover, improvement in women’s access to healthcare, smoking cessation in current smokers, later smoking initiation, wider cultural adaptation of greater involvement in sports and regular physical activity should all promote better HRQL on a populational level. We call for specifically designed research programs testing the effect of such interventions in Central Asia. With regard to occupational associations with HRQL, further research is essential to ascertain the mechanism of worse HRQL mental component in managers and secretaries.

## Conclusions

In conclusion, this is the first analysis from Central Asian countries describing non-specific HRQL in general population. Age, low income and low physical activity, all independent of each other, as opposed to occupation, are associated with poorer physical component of HRQL in this population. Moreover, being a manager, a secretary or a welder predicts poorer HRQL mental component independent of age, sex, smoking and physical activity and even each other, mandating early public health measures in prevention in the workplace. Prevention of fast HRQL degradation should not only focus on physical activity, vulnerable populations, such as women, but also on addressing low income issues and poverty in Kazakhstan. In addition, the mechanism of worse mental HRQL component in managers and secretaries should be further scrutinized, resulting in comprehensive occupational prevention programs in the workplace.

## Data Availability

The datasets used and/or analysed during the current study are available from the corresponding author on reasonable request.

## Abbreviations

BP: Bodily pain
CI: Confidence interval
GH: General health
HRQL: Health-related quality of life
MCS: Mental component score
MH: Mental health
NS: Non-significant
PCS: Physical component score
RE: Role emotional
PF: Physical functioning
RP: Role physical
SF: Social functioning
SHS: Secondhand smoke
VT: Vitality
